# Impact of the COVID-19 pandemic on predictions of death from stroke in a poor region of Brazil: a retrospective cohort study

**DOI:** 10.5935/2965-2774.20230357-en

**Published:** 2023

**Authors:** João Ricardo Bispo de Jesus, Paulo Ricardo Martins Filho, Aline Ferreira de Brito Mota, Crislaine Souza Santos, Joyce Menezes Santos, Franklim Oliveira Souza, Raphaela Barroso Guedes-Granzotti, Miburge Bolivar Gois Junior, Kelly da Silva

**Affiliations:** 1 Postgraduate Program in Applied Health Sciences, Universidade Federal de Sergipe - Lagarto (SE), Brazil; 2 Investigative Pathology Laboratory, Universidade Federal de Sergipe - Lagarto (SE), Brazil; 3 Department of Speech, Language and Hearing Sciences, Universidade Federal de Sergipe - Lagarto (SE), Brazil

Stroke and cardiovascular disease are the leading causes of morbidity and mortality worldwide. However, epidemiological indicators show a decrease in the number of stroke deaths in recent decades, which can be attributed to advances in clinical interventions. The coronavirus disease 2019 (COVID-19) pandemic and the redirection of health services raised concerns about the diagnosis and treatment of other diseases and health problems, particularly in low-income areas.^(1)^

Although the occurrence of stroke is relatively low among patients hospitalized with COVID-19, the risk of death is higher among those with these two conditions.^(2)^ Thus, the aim of this study was to investigate the impact of the COVID-19 pandemic on the prediction of deaths from stroke in a low-income region of Brazil. This was a retrospective cohort of stroke patients admitted to a tertiary hospital in the state of Sergipe between February 2019 and February 2020 (prepandemic period) and between March 2020 and March 2021 (during the pandemic). Sergipe is located in Brazil’s Northeast region, which has the highest concentration of highly vulnerable people in the country.

The following predictor variables were dichotomized: sex (female or male); age (< 60 years or = 60 years); marital status (with a partner [married, stable union, or other forms of union] or without a partner [widowed, single, or divorced]); orotracheal intubation (yes or no); alternative feeding route (yes or no); dysphagia (yes or no); poststroke motor dysfunction (yes or no); poststroke communication difficulties (yes or no); dyspnea (yes or no); mental confusion (yes or no); length of stay (< 14 days or = 14 days); stroke period (prepandemic or during the COVID-19 pandemic); and laboratory diagnosis of COVID-19 with reverse-transcriptase polymerase chain reaction (RT?PCR; yes or no). A multiple logistic regression model was built with backward selection to assess the influence of predictor variables on the outcome of interest (in-hospital death). The odds ratio (OR) with a 95% confidence interval (95%CI) was used as a measure of association. Analyses were performed by using JASP software version 0.13 (JASP Team, Amsterdam, Netherlands).

This study was approved by the Human Research Ethics Committee of the Federal University of Sergipe (approval number 4.219.456). Written informed consent was obtained from all participants.

The current study included 253 stroke patients: 115 who had a stroke before the pandemic and 138 who had a stroke during the pandemic. Most patients were men (53.8%) and over 60 years of age (82.6%). COVID-19 was identified in 20 (14.5%) of stroke patients hospitalized during the pandemic. Fifty-three in-hospital deaths were recorded, with 26 (49.1%) occurring prior to the pandemic and 27 (50.9%) occurring during the pandemic. Ten (37%) deaths reported during the pandemic were among patients infected with severe acute respiratory syndrome coronavirus 2 (SARS-CoV-2). The need for orotracheal intubation (OR = 7.01; 95%CI 2.97 - 16.55; p < 0.001) and an alternative feeding route (OR = 5.60; 95%CI 2.38 - 13.16; p < 0.001) and SARS-CoV-2 infection (OR = 4.07; 95%CI 1.26 - 13.09; p = 0.019) were associated with death among stroke patients.

This study examined how the COVID-19 pandemic affected the prediction of stroke deaths in one of Brazil’s states with the highest rates of socioeconomic vulnerability. Although some conditions, such as orotracheal intubation and the use of alternative feeding, are known prognostic factors among stroke patients, our findings point to SARS-CoV-2 infection as a new factor associated with death in this population. However, we did not observe an impact of the period of hospitalization on the occurrence of in-hospital deaths from stroke, possibly due to the continued care of acute stroke crises despite the difficulties imposed by the pandemic in Brazil.

There is evidence that SARS-CoV-2 has neuroinvasion potential^(3)^ and that the occurrence of stroke among patients with COVID-19 is higher than that among people with influenza.^(4)^ Although the exact mechanisms underlying the link between SARS-CoV-2 infection and stroke are unknown, it has been suggested that coagulopathy and endotheliopathy caused by the cytokine storm may play a role in the pathophysiology of stroke in COVID-19 patients.^(5)^ Furthermore, the occurrence of atypical and isolated neurological symptoms in COVID-19 patients has been demonstrated,^(6)^ implying the need to investigate the presence of SARS-CoV-2 infection in patients admitted with suspected stroke, particularly in regions with greater health disparities. Despite this study’s inherent limitations, our findings support the existing evidence that SARS-CoV-2 infection is a new factor associated with death among stroke patients.

## Figures and Tables

**Table 1:** Univariate and multivariate analyses of factors associated with death among hospitalized stroke patients

Variables	n	Deaths n(%)	Univariate analysis		Multivariate analysis (final model)	
			OR (95%CI)	p value	OR (95%CI)	p value
Sex						
Male	136	27 (19.9)	1.15 (0.63 - 2.11)	0.644		
Female	117	26 (22.2)				
Age (years)						
< 60	44	7 (15.9)	1.49 (0.62 - 3.57)	0.369		
= 60	209	46 (22.0)				
Marital status						
Without a partner	147	31 (21.1)	0.98 (0.53 - 1.81)	0.949		
With a partner	106	22 (20.8)				
Orotracheal intubation						
No	207	27 (13.0)	8.67(4.26 - 17.62)	< 0.001	7.01 (2.97 - 16.55)	< 0.001
Yes	46	26 (56.5)				
Length of stay (days)						
< 14	179	40 (22.4)	0.74 (0.37 - 1.48)	0.397		
= 14	74	13 (17.6)				
Alternative feeding route						
No	128	9 (7.0)	7.18 (3.32 - 15.52)	< 0.001	5.60 (2.38 - 13.16)	< 0.001
Yes	125	44 (35.2)				
Poststroke motor dysfunction						
No	83	23 (27.7)	0.56 (0.30 - 1.04)	0.067		
Yes	170	30 (17.6)				
Poststroke communication difficulties						
No	112	28 (25.0)	0.65 (0.35 - 1.19)	0.160		
Yes	141	25 (17.7)				
Mental confusion						
No	192	37 (19.3)	1.49 (0.76 - 2.92)	0.246		
Yes	61	16 (26.2)				
Stroke period						
Before the pandemic	115	26 (22.6)	0.833 (0.45 - 1.53)	0.554		
During the pandemic	138	27 (19.6)				
SARS-CoV-2 infection						
No	233	43 (18.5)	4.42 (1.73 - 11.23)	0.002	4.07 (1.26 - 13.09)	0.019
Yes	20	10 (50.0)				

OR - odds ratio; 95%CI - 95% confidence interval.
